# The Role of Neuromuscular Control of Postural and Core Stability in Functional Movement and Athlete Performance

**DOI:** 10.3389/fphys.2022.796097

**Published:** 2022-02-24

**Authors:** Erika Zemková, Ludmila Zapletalová

**Affiliations:** ^1^Department of Biological and Medical Sciences, Faculty of Physical Education and Sport, Comenius University in Bratislava, Bratislava, Slovakia; ^2^Sports Technology Institute, Faculty of Electrical Engineering and Information Technology, Slovak University of Technology, Bratislava, Slovakia; ^3^Faculty of Health Sciences, University of Ss. Cyril and Methodius in Trnava, Trnava, Slovakia

**Keywords:** body balance, core stabilization, neuromuscular functions, spinal stability, sport-specific performance

## Abstract

Balance and core stabilization exercises have often been associated with improved athlete performance and/or decreased incidence of injuries. While these exercises seem to be efficient in the prevention of injuries, there is insufficient evidence regarding their role in sport-specific performance and related functional movements. The aim of this scoping review is (1) to map the literature that investigates whether currently available variables of postural and core stability are functionally related to athlete performance in sports with high demands on body balance and spinal posture and (2) to identify gaps in the literature and suggest further research on this topic. The literature search conducted on MEDLINE, Scopus, Web of Science, PubMed, and Cochrane Library databases was completed by Google Scholar, SpringerLink, and Elsevier. Altogether 21 articles met the inclusion criteria. Findings revealed that postural stability plays an important role in performance in archery, biathlon, gymnastics, shooting, and team sports (e.g., basketball, hockey, soccer, tennis). Also core stability and strength represent an integral part of athlete performance in sports based on lifting tasks and trunk rotations. Variables of these abilities are associated with performance-related skills in cricket, cycling, running, and team sports (e.g., baseball, football, hockey, netball, soccer, tennis). Better neuromuscular control of postural and core stability contribute to more efficient functional movements specific to particular sports. Training programs incorporating general and sport-specific exercises that involve the use of postural and core muscles showed an improvement of body balance, back muscle strength, and endurance. However, there is controversy about whether the improvement in these abilities is translated into athletic performance. There is still a lack of research investigating the relationship of body balance and stability of the core with sport-specific performance. In particular, corresponding variables should be better specified in relation to functional movements in sports with high demands on postural and core stability. Identifying the relationship of passive, active, and neural mechanisms underlying balance control and spinal posture with athlete performance would provide a basis for a multifaced approach in designing training and testing tools addressing postural and core stability in athletes under sport-specific conditions.

## Introduction

Postural and core stability is critical to almost all movements in sport (Sharrock et al., 2011), particularly when maintaining balance on an uneven surface or while responding to sudden perturbations (Zazulak et al., 2007). While most research has been devoted to the role of postural stability in athletic performance, far fewer studies have investigated the relationship between core stability and sport-specific skills.

The core that involves lumbopelvic–hip region maintains the vertebral column equilibrium within its physiological limit by reducing postural displacement after unexpected perturbations (Reeves et al., 2007). This requires instantaneous activation of the central nervous system to evoke optimal muscle recruitment for both stability and mobility. Core muscles provide the necessary stability for the production of force in the lower limbs and efficient control of body movements (Rivera, 2016). Deficiencies or imbalances in the core muscles can increase fatigue, decrease endurance, and increase the risk of injuries in athletes (Rivera, 2016).

Recently, widely promoted spine stabilization and core strengthening exercises have been seen to improve postural and core stability and/or reduce back problems in athletes (Akuthota et al., 2008). These exercises seem to be efficient in the prevention and rehabilitation of back pain, lumbar spine injuries, or other musculoskeletal disorders. However, there is a lack of evidence regarding their effectiveness for improvements of functional movements and consequently also athletic performance. This is mainly due to a limited number of appropriate tests evaluating postural and core stability that would be able to provide deeper insights into the understanding of exercise-induced changes in neuromuscular functions under sport-specific conditions.

Currently, motion analysis and accelerometry recordings allow monitoring of head, trunk, and limb movements and provide useful data for a complete assessment of postural and core stability during a variety of functional movements. Measurement of postural sway using accelerometry is strongly related to task-based variables (Whitney et al., 2011). The accelerometry combined with stochastic dynamics quantifies the time-varying structure of postural sway pattern (Lamoth et al., 2009). For instance, acceleration time-series are more stable, less variable, and less regular with greater gymnastic skills (Lamoth et al., 2009).

In the study by Glofcheskie and Brown (2017), a seated balance task was used to assess trunk postural control, electromyography, and kinematics to measure neuromuscular control in response to unexpected trunk perturbations, and active trunk repositioning tasks to examine proprioceptive ability. There was an interactive relationship between postural control, trunk neuromuscular control, and trunk proprioception in athletes of different training backgrounds (Glofcheskie and Brown, 2017). More specifically, greater trunk postural control (less CoP movement), less lumbar spine angular displacement, higher muscle activation amplitudes, and faster trunk muscle activation onsets in response to unexpected trunk perturbations were found in athletes (collegiate level long-distance runners and golfers) than non-athletes (Glofcheskie and Brown, 2017). Absolute and variable errors in trunk repositioning tasks were lower in golfers than runners and controls, which indicates their greater proprioceptive ability (Glofcheskie and Brown, 2017).

Usually, postural and core stability have been compared among athletes of different sports, their age, and/or performance level. For instance, the best body balance is found in gymnasts, then in soccer players, swimmers, physically active controls, and basketball players (Hrysomallis, 2011). Balance is related to the competition level of athletes, and the more proficient ones display better postural stability (Hrysomallis, 2011). Athletes of rifle shooting, soccer, and golf have better postural stability than their less-proficient counterparts (Hrysomallis, 2011). Paillard (2019) reported that the most successful athletes have the best postural performance, both in ecological and non-ecological postural conditions, that is, specific vs. decontextualized in relation to the sport practiced. They also have more elaborate postural strategies than those at lower competition levels (Paillard, 2019). Specific muscle synergies are of considerable value as a training strategy for hockey players who need to improve their postural stability and reduce their potential risk of injuries (Kim et al., 2018).

Balance is also associated with performance measures (Hrysomallis, 2011). Body sway measured during stance on a force plate is related to aim point fluctuation and shooting performance (Ball et al., 2003). As body sway increases, performance decreases and aim point fluctuation increases for most relationships in elite rifle shooters (Ball et al., 2003). Postural balance in the standing position is also related to the shooting accuracy, both directly and indirectly, through rifle stability (Mononen et al., 2007). Furthermore, a balance ratio (contact with floor to no contact time) during a 30-s wobble board test correlates with maximum skating speed in hockey players (Behm et al., 2005). Unipedal static balance, core strength, and stability correlate with golf performance in elite players (Wells et al., 2009). There is also a relationship between unipedal dynamic balance and the luge starting speed (Platzer et al., 2009a).

In general, practicing any kind of sport is associated with better postural stability (Andreeva et al., 2021). The center of pressure (CoP) velocity during a bipedal stance on a force platform with eyes open is lower in shooters, football players, boxers, cross-country skiers, gymnasts, runners, team sport players, wrestlers, tennis players, alpine skiers, rowers, speed skaters, and figure skaters when compared to the general population (Andreeva et al., 2021). Athletes usually display better postural stability in sport-specific conditions and sway measures may not reveal between and within-group differences when testing in a standard upright position (Zemková, 2014). There are also differences in the magnitude of postural sway increase after sport-specific exercises and the speed of its readjustment to pre-exercise level (Zemková, 2014).

Investigating the relationship of passive, active, and neural mechanisms underlying balance control and spine stabilization with sport-specific performance would provide a basis for a multifaced approach in designing training and testing tools addressing postural and core stability in athletes. The aim of this scoping review was (1) to review the existing literature that deals with sports with high demands on body balance and spinal posture and to investigate whether currently available variables of postural and core stability are functionally related to athletic performance and (2) to identify gaps in the literature and suggest further research on this topic.

## Methods

This article was proposed as a scoping review (Armstrong et al., 2011). The purpose was to provide an overview of the available research evidence and answer the following question: (1) Is there a relationship between postural and core stability and functional movement and/or athletic performance?

An electronic literature search was provided to analyze existing studies dealing with the role of neuromuscular control of both postural and core stability in functional movement and/or athlete performance. Studies were searched on Scopus, Web of Science, PubMed, MEDLINE, and Cochrane Library databases. This search was completed on Google Scholar, Elsevier, and SpringerLink. The articles in peer-reviewed journals were considered for analysis. References included in review articles were also searched to identify further relevant studies. If articles included overlapping data from the same or similar study, the one with the most recent publication date was analyzed. Articles or abstracts published in conference proceedings, theses, case studies, and books were excluded. Articles were also excluded if they did not contain original research or were incomplete. The inclusion criteria involved research articles that specified participants, experimental protocols, and measures relevant to this review. The literature search was limited to the English language. Articles published after 1990 were preferred. Articles were excluded if they failed to meet the eligibility criteria.

The initial search was confined to research articles closely related to the main purpose of this scoping review, that is, those dealing with the relationship between neuromuscular control of either postural or core stability and functional movement and/or athlete performance. However, this approach revealed only a limited number of articles that met the eligibility criteria. The search was, therefore, widened to investigations dealing with the effects of sport-specific and balance or the core-related exercises on functional movements and skills within a particular sport. In particular, neuromuscular mechanisms underlying these relationships were studied. This together helped us to identify gaps in the literature and formulate recommendations for further studies in this field of research.

The search and appraisal of selected studies on the basis of exclusion and inclusion criteria were performed by both authors of this review. Some concerns were related to sample size and its representativeness, incomplete information about the methods used, variables analyzed, and/or non-controlled compliance of experiments. The target population was athletes of a team and individual sports where balance and core stability play an essential role in their performance. Proposed sports were combined with the following keywords.

A combination of these terms was included in the search strategy: “postural stability” AND “core stability” AND “core endurance” AND “core strength” AND “core training” AND “body balance” AND “postural control” AND “spinal posture” AND “lumbopelvical stability” AND “athletes” AND “sport-specific exercise” AND “athletic performance” AND “functional movement,” AND “neuromuscular control.”

**FIGURE 1:**
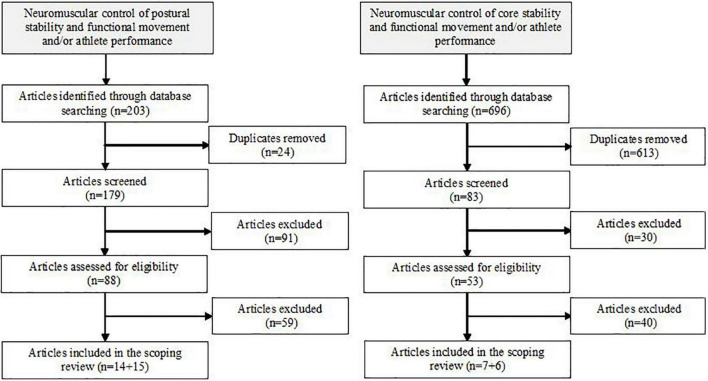
Flow chart showing the literature search phases.

Further searches were conducted by using words from subheadings that specified the contribution of postural and core stability on performance in highly skilled athletes in comparison with those at a lower level of sport-specific skills. Following an initial screening of articles identified through database searching and assessing for their eligibility, those that failed to meet inclusion criteria were removed. Articles that investigated neuromuscular control of postural (14 out of 29) and core (7 out of 13) stability in association with functional movements and/or athlete performance were included in this scoping review. The search process phases are displayed in Figure 1.

## Results and Discussion

### The Role of Neuromuscular Control of Postural Stability in Functional Movement and/or Athlete Performance

Analysis of the literature revealed (Table 1) that postural stability plays an important role in functional movements and/or athlete performance in shooting (Era et al., 1996; Ball et al., 2003; Mononen et al., 2007; Ihalainen et al., 2016a,b, 2018; Lang and Zhou, 2021), gymnastics (Opala-Berdzik et al., 2021), dancing (Munzert et al., 2019), and team sports, such as soccer (Jadczak et al., 2019a,b).

**TABLE 1:** Neuromuscular control of postural stability and functional movement and/or athlete performance.

Authors	Study design	Participants	Main variables	Main findings	Postural stability control and athlete performance
**Relationship between postural stability and functional movement and/or athletic performance**					
Ball et al., 2003	20 shots under competition conditions	6 elite shooters	AMTI LG6-4 force plate for measuring body sway parameters; SCATT shooting analysis system for measuring aim point fluctuation and shooting performance	Body sway is related to performance in shooters; Body sway is related to aim point fluctuation in shooters; As body sway increases, performance decreases and aim point fluctuation increases for most relationships	Body sway and aim point fluctuation are essential in elite rifle shooting; Highly individual-specific are performance errors
Behm et al., 2005	Relationships between hockey skating speed and specific performance measures	30 competitive junior and secondary school hockey players	Off-ice measures including squat jump, drop jump, a 40-yd sprint, 1 RM leg press, flexibility, and balance ratio; Electromyographic (EMG) activity of the dominant vastus lateralis and biceps femoris while skating, performing a change-of-direction drill, stopping, and turning	There are significant correlations between sprint and balance tests and the skating performance; There are significant correlations between balance and players under the age of 19 years but not those over 19 years old	Significant correlations with balance suggest that stability may be associated with skating speed in younger players; Low correlations with drop jumps suggest that short contact time stretch-shortening activities is not an essential factor; EMG activities illustrate very high activation levels associated with maximum skating speed
Mononen et al., 2007	30 shots in the standing position at a distance of 10 m from the target	58 right-hand male conscripts from the Finnish Air Force Communications School	Postural balance and rifle stability assessed in terms of anteroposterior and mediolateral sway velocity of the CoP movement, and horizontal and vertical deviation of the aiming point	Shooting accuracy is related to postural balance and rifle stability, but only at the inter-individual level; There is a correlation between shooting score and behavioral performance variables; Postural balance is related to the shooting accuracy both directly and indirectly through rifle stability	High postural balance and minimal movement of the gun barrel are essential determinants of successful shooting performance among novice shooters
Edıs et al., 2016	Relationships between postural control variables and technical performance in different small-sided games (SSGs) - 1:1, 2:2 and 3:3	16 trained male amateur soccer players	Measuring of postural sway in anterior–posterior and medial–lateral directions during one-legged and both-legged quiet-stance using a Tekscan HR Mat™	There is a relationship between postural control and soccer-specific technical variables in 1:1, 2:2 and 3:3 SSGs	Higher postural control levels are essential variables that affect success in technical skills under rival pressure and suddenly changing conditions
Ihalainen et al., 2016a	A simulated air rifle shooting competition series	40 international- and national-level shooters	Optoelectronic device for measuring of shooting score and aiming point trajectory variables; Force platform for measuring of postural balance variables	Stability of hold, cleanness of triggering, timing of triggering and aiming accuracy are predictors of shooting performance, accounting for 81% of the variance in a shooting score; Direct effect of postural balance on performance is small, accounting for <1% of the variance in a shooting score; Indirect effects could be greater through a more stable holding ability that correlate with postural balance	Aiming accuracy, cleanness of triggering, and timing of triggering contribute to shooting score in elite-level air rifle shooters
Ihalainen et al., 2016b	Simulated series of air-rifle shooting-competition in three consecutive seasons	17 elite shooters	Optoelectronic shooting device for measuring of shooting score and aiming-point-trajectory variables; Force platform for measuring of postural-balance variables	Seasonal mean test results in stability of hold and cleanness of triggering are related to competition performances; Changes in stability of hold and cleanness of triggering are related to changes in performances; Cleanness of triggering is related to postural balance in shooting direction, whereas stability of hold is related to balance in cross-shooting direction	Stability of hold, cleanness of triggering, and postural balance affect performance in both training and competition situations in athletes at the elite level
Verhoeven and Newell, 2016	50 basketball free-throws with both their dominant and non-dominant hand	25 male college students with a range of skill levels	The free-throw shot recorded at 120 Hz through 8 VICON Bonita Optical motion capture cameras	Trial-to-trial variance in release parameters as well as postural stability of the shooter, synchronization of postural movement and ball release are strong predictors of performance, with non-elite shooters having a higher mean and variability of CoM speed at the time of ball release; The synchronization between the time of peak CoM and the time of ball release increases as a function of skill level and hand dominance, with the better performers releasing the ball more closely to the time of CoM peak height	The control of the trial-to-trial variability along the solution manifold of release parameters, as well as the coordination of postural control and ball release properties are important for shooting success changes as a function of skill level
Edıs et al., 2017	Relationships between parameters designating postural control levels and running speeds in SSG	16 youth soccer players	Measuring of postural sway in anterior-posterior and medial-lateral directions during one and both leg stance positions	There is a significant relationship between the running speeds of 0–6, 6–10 and 10–16 km.h^–1^ in 2 vs. 2 and 3 vs. 3 games	Combining practices that are designed to train balance with football specific exercises in a single training session can significantly contribute to athlete performance
Spratford and Campbell, 2017	The effect of postural stability pre- and post-arrow release, arrow length, flight time, draw force and clicker reaction time on scoring outcomes and the performance	39 recurve archers of an elite-level (23 male and 16 female) from four different countries prior to competition at a World Cup event	The CoP measurements 1 s prior to arrow release and 0.5 s post-arrow release using an AMTI force platform (1000 Hz); High-speed footage (200 Hz) for calculation of arrow flight time and flight score	Maximum sway speed, draw force and clicker reaction time are variables that predict performance of the shot; Higher bow draw force, reduced clicker reaction time and postural sway speed post-arrow release are predictors of higher scoring shots	The clicker time, draw force and mainly maximum sway speed post-arrow release play an important role in the scoring outcomes in elite-level recurve archery
Ihalainen et al., 2018	Factors determining performance in biathlon standing shooting at rest as well as after intense exercise	9 junior 8 national team biathletes	40 resting shots (REST) and 2 × 5 shots simulating the competition (LOAD) after 5 min of roller skiing at 95% of peak heart rate; Postural balance, aiming point trajectory and hit percentage measured from each shot	Cleanness of triggering (ATV) and vertical stability of hold (DevY) are the most important components affecting shooting performance both in REST and in LOAD; Postural balance, especially in shooting direction, is related to DevY and ATV	Cleanness of triggering and vertical holding ability are key factors in biathlon standing shooting performance; Postural balance especially in shooting direction is related to these shooting technical components; Athletes may be able to reduce the movement of the aiming point in triggering phase and in the holding phase by improving their postural stability
Caballero et al., 2021	Relationships between balance and tennis performance using linear and non-linear parameters through (1) the comparison of tennis players of different levels of expertise and ages and (2) the analysis of the association of balance and tennis serving speed and accuracy	106 recreational and expert male tennis players	Temporal dynamics of postural control during a balance task on an unstable surface analyzed through the mean velocity and the detrended fluctuation analysis (DFAV) of CoP	Traditional variables measuring balance performance only show differences according to age but not to sport performance; CoP show a reduction of auto-correlated variability with age in expert players; CoP dynamics is related to age and discriminates sport expertise	Sport experience induces balance adaptations that is characterized by a higher ability to perform postural adjustments; The lack of correlations indicates that balance measured with scattering variables under non-specific conditions is not a determinant of tennis serve performance
Lang and Zhou, 2021	60 shots under test conditions	12 elite athletes belonging to national team	Shooting score for indicating of performance; Footscan 1.0 force platform for measuring of postural balance variables; SCATT MX-02 optoelectronic training device for measuring of aiming technique parameters	Postural balance is negatively correlated with shooting score and aiming accuracy; Postural balance is positively correlated with the stability of hold and stability of triggering; There is a significant correlation between postural balance and performance, aiming accuracy and stability of hold for shooters; Postural balance is related to the stability of triggering for shooters; Postural balance is not significant with aiming time on an intra- and inter-individual basis	Postural balance is very important in aiming technique and shooting performance among elite rifle shooters
Opala-Berdzik et al., 2021	Differences in postural steadiness among young gymnasts practicing different disciplines, and their relation to the duration of their training experience, age, and their anthropometric characteristics	10 female artistic gymnasts, 10 female acrobatic gymnasts, and 10 female non-athletes	60-s quiet standing trials on a force platform with the eyes open and closed; Postural sway represented by directional components of CoP mean velocity	There are no differences in anterior-posterior (A-P) and medial-lateral (M-L) CoP mean velocities between the acrobatic and artistic gymnasts; The age, body height, body mass, duration of training experience, and maturity offset are negatively correlated with the A-P CoP mean velocity under eyes-open conditions in the artistic gymnasts; The body mass and BMI percentiles are negatively correlated with A-P and M-L CoP mean velocities under both visual conditions in the acrobatic gymnasts; The non-athletes’ CoP mean velocities are non-significantly correlated with their age and anthropometric measures under both visual conditions	The artistic gymnasts’ longer training experience, greater age, body height, body mass, and biological maturity are associated with better anterior-posterior postural steadiness when vision is available; The acrobatic gymnasts’ greater body mass and BMI percentiles are associated with better overall postural steadiness regardless of visual conditions; There are relationships between postural steadiness and discipline-specific training experience and anthropometric characteristics
Sarro et al., 2021	The relationship between bow stability and postural control in recurve archery according to shooting performance	8 archers who participated in national-level competitions, and trained four times a week	6 shot of arrows at a 13-m distant target; 3-dimensional position of one marker attached to the bow and the CoP position of the archer measured during the aiming phase, representing bow and archer displacement, respectively	Length of the CoP trajectory (DCoP), CoP displacement in the direction across the target (CoPY), and length of the bow trajectory (Dbow) are higher in the lowest than the highest scoring shot; There is a significant correlation between CoPX and vertical displacement of the bow (DZ) during the highest scoring shot, and between CoP and bow displacement in the direction towards/away from the target (CoPX and DX)	Synchronization between body and bow sway may influence the accuracy of the shot, suggesting that combined balance and bow stability training exercises would be beneficial to improve archery performance
**Effect of sport-specific training on functional movement and/or athletic performance**					
Larue et al., 1989	The stability of body-gun up to the firing of a shot when shooting in standing position	2 novices and 2 experts in rifle shooting, 2 novices and 2 experts in biathlon	Electromyographic activity of the tibialis anterior, gastrocnemius and deltoid muscles; Rifle oscillation and CoP displacements	Expert biathletes use a different strategy than expert rifle shooters; No significant pattern emerges from tests with novice rifle shooters	Rifle shooters and biathletes adapt characteristics of their disciplines
Aalto et al., 1990	A simulated race	10 competition shooters	Force platform for measuring postural stability with and without competition clothing	Stability is significantly better in shooters than in untrained controls without supportive clothing; The Romberg quotient is higher in shooters than in controls, suggesting that they use an increased amount of vestibular and proprioceptive cues to stabilize their posture	Assiduous training aimed to improve balance contributes to good postural stability in shooters
Era et al., 1996	Posture control while aiming 7.5 s preceding the shot	National top-level, national and amateur rifle shooters	Speed and amplitude of the center of forces (CoF) movement	Top-level male shooters stabilize their posture better than top-level female and national level male shooters, who are more stable than naive shooters; Experienced shooters stabilize their posture better during the last seconds preceding the shot, whereas there are no differences when the successive windows are compared with each other in naive shooters; Naive shooters have more pronounced CoF movement in the less successful trials; Not-efficient whole-body posture stabilization is not a reason for a poor result in top-level shooters	Postural control is better in trained athletes who can improve their stability during the last seconds preceding the shot; A good body stabilization is the prerequisite for good shooting performance
Bringoux et al., 2000	How motor skills experts requiring a good postural control perceive their body orientation with few gravity based sensory cues	5 expert gymnasts (4 males and 1 female) and 5 non-gymnast subjects (3 males and 2 females)	The body tilt when pitching at 0.05 deg.s^–1^ in conditions of body restriction (strapped and body cast altering the somatosensory cues); The Subjective Postural Vertical (SPV) starting from different angles of pitch tilt	There is a larger body tilt when totally restrained in the body cast in controls than in experts; Controls exhibit significant errors of SPV judgmentwhereas the experts are very precise	More informative are somatosensory than otolithic cues for the body orientation perception; The efficiency of otolithic and/or interoceptive inputs can be improved through a specific training to compensate for the lack of somatosensory cues
Asseman et al., 2004	Comparing the level of performance and postural control of elite gymnasts in postures specifically trained or not	15 elite gymnasts	Surface and mean velocity of the CoP motions	The subject’s level of postural performance and control in one condition is not correlated to the corresponded level in another condition; Postural ability of elite gymnasts in the handstand is not transferable to upright standing postures	Body movements and muscular force regulation to maintain balance are specific to the task characteristics; Postural performance and control in gymnastics’ skills are not transferred or generalized to more usual upright stances; The level of athlete in this activity does not implicate a corresponding level in usual postures
Asseman et al., 2008	A comparison of body sway in bipedal and unipedal with eyes open and eyes closed	Two groups of 13 subjects: male elite gymnasts of international level and portsmen of regional level involved in different activities	Center of gravity motion computed from CoP motion, estimating postural control	The two groups differ significantly in the unipedal posture and with eyes open; Removal of vision affects similarly both groups	Gymnastics expertise improves postural performances in situations for which their practise is related to (i.e., unipedal with eyes open)
Croix et al., 2010	The effect of somatosensory and visual information on handstand performance; The link of general perceptual characteristics of gymnasts with their handstand performances	17 gymnasts: an expert group (6 women and 2 men), and a non-expert group (7 women and 2 men)	A handstand on a force platform in 4 conditions: open or closed eyes on a firm or foam support; The surface area covered by the CoP trajectory	Experts have significantly better postural performance during the handstand than nonexperts, whatever the visual condition, nonexperts are unable to maintain the handstand without vision, whatever the support, and the CoP surface is significantly greater on the foam surface than on the firm surface for both experts and nonexperts and, only for experts, whatever the visual condition; Experts are less field dependent than nonexperts, and the rod-and-frame test results are positively correlated with postural performance	Expert gymnasts use the remaining sensory modalities efficiently when vision is removed; Gymnastics training improves the ability to change the frame of reference
Butler et al., 2016	Differences in dynamic balance across competition levels in baseball players	90 professional (PRO), 78 collegiate (COL), and 88 high school (HS) baseball players	Lower Quarter Y-Balance Test	The PRO players exhibit greater posteromedial reach, posterolateral reach, and composite score than HS and COL groups; HS baseball players exhibit increased anterior reach compared with the COL and PRO cohorts; There are no significant differences in reach asymmetry among groups	Baseball players of different competition level differ in lower extremity dynamic balance performance
Omorczyk et al., 2018	Relationships between stability indices registered in two positions	46 athletes (23 juniors and 23 seniors) practicing gymnastics at various levels of advancement	Standing and handstand; Posturograph CQ-Stab 2P	CoP area, mean CoP amplitude, mean CoP displacement of the feet/hands in M-L direction and maximal CoP displacement of the feet/hands in M-L direction in both standing position and handstand is significantly lower in seniors; The statokinesiogram path length, both total and in A-P and M-L directions in the standing position is significantly lower in seniors	Ability to control the position of the body in both positions is better in seniors than in juniors; Stability variables in standing position significantly correlate with those in handstand in seniors but not in juniors
Jadczak et al., 2019a	Body balance control and recovery strategies in static and dynamic conditions	Professional and junior elite soccer players: 52 in PRO, 55 in U-21, 47 in U-19 group	Body balance control measured using a Delos Postural Proprioceptive System	Static and dynamic balance varies among players in different playing positions; Significantly higher differences in the static test with eyes closed are in central midfielders than goalkeepers; There is a difference in the dynamic postural priority test in favor of central midfielders relative to external defenders, central defenders, external midfielders and forward; The difference is greater in non-dominant than dominant leg	The higher the sport level of football players (PRO), the better their balance, which may contribute to more effective performance of actions related to the game and the prevention of injuries
Jadczak et al., 2019b	A comparison of balance profiles in elite soccer players across different field positions	101 elite professional soccer players (10 goalkeepers, 15 central defenders, 15 external defenders, 23 central midfielders, 15 external midfielders, 23 forwards)	Delos Postural System Test using the standard protocol (standing on a stable platform and on an unstable base unilaterally on non-dominant and dominant leg with eyes open and eyes closed)	Central midfielders have significantly higher differences than goalkeepers in the static test with eyes closed; There is a difference in favor of central midfielders relative to external defenders, central defenders, external midfielders and forwards in the dynamic postural priority test; There is a significantly greater difference in non-dominant compared to dominant leg in the dynamic postural priority test	Static balance performance and dynamic postural priority varies with playing position in elite soccer players; Midfield players have better postural priority than players in other positions; Professional soccer players present greater balance postural priority on the non-dominant leg
Marcolin et al., 2019	The effect of training on postural control in sport-specific and simple tasks	Eight female advanced-level gymnasts (ALG) and seven female basic-level gymnasts (BLG)	Bipedal standing (B) and single-leg back scale (BS) before and after two gymnastic elements (rondade plus flic-flac)	Better postural control in the B position in BLG, whereas in the BS position in ALG; CoP parameters increase after the rondade plus flic-flac in both BLG and ALG; Better performance on balance time-dependent response after the rondade plus flic-flac in BS in ALG	Postural control during the simple task (B) is not affected by expertise level; The sport-specific task (BS) is more selective in representing the level of expertise in young gymnasts
Munzert et al., 2019	Expertise-specific differences in postural tasks of various difficulty	12 intermediate non-professional and 13 professional dancers	Five dynamic dance-like and Six static everyday postural tasks	There is an expert advantage on sway area for dance-like but not for static everyday postural tasks; This effect is observed for the root mean square (RMS) velocity and RMS amplitude of the difference signal between CoP and CoG line location	The expert advantage is task-specific and provide new insights into the specificity of postural performance in experts
Caballero et al., 2020	The relationship between team-handball performance and balance ability according to expertise and age, applying a non-linear approach to balance assessment	114 male team-handball players	The CoP during a balance task; Sport performance measured by the speed and accuracy in throwing	There is a faster but not more accurate throw in expert than recreational players; Balance performance is better for 18+ than U12 players, whereas there are no differences according to their skill level; CoP velocity is slower during the balance task and moves are less irregular in players who throw with less accuracy; CoP movements are more irregular and less auto-correlated in players who throw faster	Balance performance is better in experienced team-handball players, and this is related to the maturation of the motor system more than to sport performance level; There is an exploratory behavior during balance in expert players, exhibiting more motion adjustments to reduce motor output error; A non-linear assessment reveals functional variability of balance as an intrinsic characteristic of individuals’ motor control according to skill level and age
Andreeva et al., 2021	Postural stability in athletes of various sports	936 athletes of different sports and performance level	The CoP sway area (AS) and velocity (VCP) during bipedal stance with eyes open (EO) and eyes closed (EC) on a stabiloplatform (50 Hz)	VCP-EO increases in shooters, footbal players, boxers, cross-country skiers, gymnasts, runers, team sport players, wrestlers, tennis players, alpine skiers, rowers, speed skaters, and figure skaters compared to controls	Practicing sport is associated with increased postural stability in bipedal stance

#### Shooting, Biathlon, and Archery

The majority of studies investigated postural stability in association with shooter performance. Postural stability and stability of hold were identified as the main factors influencing air rifle shooting performance (Era et al., 1996; Konttinen et al., 1998; Ball et al., 2003). High postural stability and small gun barrel movements determine shooting performance in novice shooters (Mononen et al., 2007). High postural stability is also important in elite rifle shooters (Lang and Zhou, 2021). Specifically, CoP variables measured during stance on a force plate negatively correlate with shooting score and aiming accuracy, whereas there is a positive correlation with the stability of hold and stability of triggering (Lang and Zhou, 2021). The timing of triggering, cleanness of triggering, and aiming accuracy then influence shooting score in elite-level air rifle shooters (Ihalainen et al., 2016a). Taking together, postural stability, cleanness of triggering, aiming accuracy, and stability of hold affect performance in both training and competition situations even in athletes at high–shooting skill levels (Ihalainen et al., 2016b). Particularly, body sway is related to aim point fluctuation in shooters (Ball et al., 2003). Aim point fluctuation increases and performance decreases as body sway increases for most relationships (Ball et al., 2003). However, Spancken et al. (2021) identified that body sway does affect shot score in national- and elite-level athletes in both small-bore and air-rifle shooting, whereas aiming time, aiming accuracy, and horizontal rifle stability influence shot score in national-level air-rifle athletes. A higher Romberg quotient in shooters than in controls indicates that they use an increased amount of vestibular and proprioceptive cues to stabilize their posture (Aalto et al., 1990). The coordination patterns of pistol motion and posture are more variable in the novice than in the skilled group (Ko et al., 2018). There are different quantitative and qualitative dynamics in pistol-aiming reflecting athlete’s skill level with postural control foundation (Ko et al., 2018). The skill acquisition in pistol-aiming reduces the kinematic variables into a lower-dimensional functional unit over the posture and upper-limb system (Ko et al., 2017).

Vertical holding ability and cleanness of triggering are also important for shooting performance in the biathlon (Ihalainen et al., 2018). Postural stability in shooting direction is related to technical components of shooting, which indicates that athletes can reduce the aiming point movement in the holding and triggering phase by stabilizing their posture (Ihalainen et al., 2018). It seems that expert biathletes use a different strategy than expert rifle shooters, each of them adapting to the characteristics of their respective discipline (Larue et al., 1989).

The synchronization of bow and body sway plays a role in shot accuracy, which indicates that combined bow stability and balance exercises would contribute to better archery performance (Sarro et al., 2021). Reduced postural sway speed post-arrow release, greater bow draw force, and reduced clicker reaction time are predictors of higher scoring shots in elite recurve archers (Spratford and Campbell, 2017).

#### Gymnastics

Furthermore, specific postural stability control plays an essential role in acrobatic sports. Gymnastic experience during childhood is beneficial for the development of proprioceptive reweighting processes that lead to a more mature form of controlling and coordinating posture similar to adults (Busquets et al., 2021). Anthropometric characteristics and discipline-specific training experience are associated with postural steadiness (Opala-Berdzik et al., 2021). There is a relationship between anterior–posterior postural steadiness with eyes open and the artistic gymnasts’ biological maturity, body mass, body height, greater age, and longer training experience (Opala-Berdzik et al., 2021). Overall postural steadiness regardless of visual conditions is associated with the acrobatic gymnasts’ BMI percentiles and greater body mass (Opala-Berdzik et al., 2021).

The sport-specific task (i.e., single-leg back scale) is more sensitive in differentiating the level of expertise in young gymnasts than the simple task (i.e., bipedal standing) (Marcolin et al., 2019). While basic-level gymnasts have better postural stability in the bipedal standing, advanced-level gymnasts perform better in the single-leg back scale, particularly on balance time-dependent response to the rondade plus flic–flac (Marcolin et al., 2019). In addition, experts have a more efficient perception of body orientation in space in skills that require a fine postural adjustment than controls (Bringoux et al., 2000). Increasing expertise through specific gymnastic training increases the relevance of interoceptive and/or otolithic inputs (Bringoux et al., 2000). There is an expert advantage on sway areas for dance-like but not static postural tasks (Munzert et al., 2019). Their advantage is task specific, which provides new insights into the specificity of postural performance in highly skilled athletes (Munzert et al., 2019).

Specific postural control in gymnasts’ skills (i.e., postural ability in the handstand) is not transferred to basic upright stances (Asseman et al., 2004). Gymnastics expertise seems to improve postural performance only in conditions to which their practice is related (Asseman et al., 2008). There is lower frequency and variation of body sway in the handstand in more than less-experienced gymnasts (Sobera et al., 2019). While the first group concentrates on reducing anterior–posterior body sway with minimum medial–lateral movements, its values in a medial–lateral direction are irregular in the second group (Sobera et al., 2019). Postural performance in the handstand is significantly better in expert gymnasts than non-experts, whatever the visual condition (Croix et al., 2010). Experts are less field-dependent than non-experts, and this is positively correlated with postural performance (Croix et al., 2010). They use the remaining sensory modalities more efficiently under eyes closed conditions (Croix et al., 2010). The ability to change the frame of reference is improved through a high level of gymnastics training (Croix et al., 2010). Variables obtained in the handstand and standing position significantly correlate in the senior but not in the junior gymnasts (Omorczyk et al., 2018). Disabling visual control in the handstand and free-standing position in seniors deteriorates postural sway and increases CoP displacement in the anteroposterior and both directions. Lack of differences in CoP variables in the mediolateral direction in a free-standing position indicates that eye control is not important for body stability in the frontal plane in seniors practicing gymnastics CoP movement control in the mediolateral direction (Puszczałowska-Lizis and Omorczyk, 2019).

#### Team Sports

Postural stability control is also important for performance in team sports and may vary among athletes of different competition levels. Both dynamic and static tests should be used for the assessment of balance as postural control performance in these two cases is not related (Pau et al., 2015). For instance, the measures from the Star Excursion Balance Test may not reflect the balance performance in well-trained athletes (i.e., professional basketball and football players) who have a better balance when performing sport-related skills (Halabchi et al., 2020). However, this test includes static postures, which may better reflect postural deficits in more experienced athletes than dynamic tests (Halabchi et al., 2020).

The hockey skating performance significantly correlates with balance and sprint tests, which demonstrates the important role postural stability plays in skating speed in young players (Behm et al., 2005). Improving postural control by decreasing CoM speed at ball release is important for a higher level of shooting in basketball (Verhoeven and Newell, 2016). Incorporating postural control in the free throw shot is a critical qualitative change in coordination resulting from practice (Verhoeven and Newell, 2016). Also, volleyball players may develop a unique postural control (Borzucka et al., 2020b). Their sensory resources should be optimally distributed between sport-specific skills on the court and postural control (Borzucka et al., 2020b). They use diversified postural strategies for the maintenance of balance whereby reducing the contribution of proprioception for more challenging posture-motor tasks (Borzucka et al., 2020a). A different model of sensory integration is used by volleyball players for postural control compared to non-athletes, which may be explained by their better “dynamic” visual acuity (Agostini et al., 2013). Dynamic balance is better in professional than collegiate and high school baseball players (Butler et al., 2016).

Soccer players have superior postural control when compared to those involved in contact sport and no sport at all (Liang et al., 2019). Contact sports increase postural control through increased use of vestibular and proprioceptive information (Liang et al., 2019). Players with soccer-specific training improve executive control and proprioceptive functions, which results in better single-support balance during a dynamic visuomotor lower limb-reaching task (Snyder and Cinelli, 2020).

The contribution of vision in the maintenance of balance is less important in the professional national-level than amateur regional-level soccer players (Ben Moussa et al., 2012). Balance performance in terms of more efficient and faster stabilization after a forward jump is better in young national-level soccer players, whereas a one-leg static standing test is not sensitive enough to reveal differences in postural control associated with the combination of physical and technical features (Pau et al., 2018). Stabilometric variables improve with age until maturity (Zago et al., 2020). The higher the sport level of football players, the better their balance (Jadczak et al., 2019a). Greater balance in professional soccer players is on the non-dominant leg (Jadczak et al., 2019b). Static balance in elite soccer players varies across playing positions with better postural control in midfield players than those in other positions (Jadczak et al., 2019b). The level of playing experience influences postural control in test conditions specific to playing soccer (Paillard et al., 2006). Postural regulation changes from visual to vestibular and proprioceptive contribution, which allows better visual control of game situations in the field (Paillard et al., 2006).

Experienced team-handball players exhibit better balance performance, which is more associated with the maturation of the motor system than their performance level (Caballero et al., 2020). It seems that players with a higher level of expertise exhibit a better ability to perform motion adjustments to reduce motor output error (Caballero et al., 2020). Although postural adjustments during a balance task have a differential feature in expert players, this ability is not crucial for a tennis serve performance (Caballero et al., 2021). However, this does not reject the association of balance with other tennis drills such as pivoting maneuvers, sudden decelerations, and fast cutting maneuvers (Caballero et al., 2021).

#### Other Sports

Furthermore, balance, core strength and stability, flexibility, and peripheral muscle strength are associated with golf performance (Wells et al., 2009). Using concurrent mental tasks, differences in balance performance between expert surfers and controls can be found, whereas standard balance tests may not be able to elucidate whether surfing expertise facilitates balance adaptations (Chapman et al., 2008). When sharing attention with a concurrent mental task, sway path length increases in expert surfers compared to controls (Chapman et al., 2008). A different model of sensory integration was found in young kayaking and canoeing athletes than in non-athletes, which may be ascribed to a subtle re-adaptation deficit after disembarking to a stable surface with diminished sensitivity of vestibular apparatus and vision (Stambolieva et al., 2012). Better postural stability is also present in pentathletes who are less vision-dependent than untrained individuals. Conscious control of body alignment and a high level of concentration are the main factors responsible for minimizing body oscillations in pentathletes (Sadowska et al., 2019). Horseback riding may develop better postural muscle tone and particular proprioceptive abilities on standing posture during bipedal dynamic perturbations (Olivier et al., 2019). Interestingly, less anteroposterior movement during chair rising was found in master runners compared with young athletes, suggesting that they are not spared from the age-associated decline in postural stability and may benefit from specific balance training (Leightley et al., 2017).

However, some studies found no significant relationship between postural balance control and athlete performance. For instance, both the isokinetic core power and a one-legged static balance do not correlate with overall World Cup points in competitive snowboarders (Platzer et al., 2009b). Furthermore, unilateral stance with eyes closed demonstrates a positive correlation with pitch velocity, whereas there is no significant correlation between unilateral stance with eyes open or eyes closed and pitching error in college baseball pitchers (Marsh et al., 2004). Similarly, there is a lack of correlation between balance, measured with scattering variables in a non-specific task, and tennis serving speed and accuracy (Caballero et al., 2021). Furthermore, balance is not associated with team-handball performance (Caballero et al., 2020). Although the accuracy of the throws revealed a slight positive correlation with mean CoP velocity magnitude (players with better throw accuracy moved faster during the balance task), there was a negative correlation between the ball speed and bivariate variable error in experts (Caballero et al., 2020). Nevertheless, low-to-moderate correlations between unipedal balance ability and the players’ technical level suggest that some technical soccer skills improve more after balance than typical soccer training (Cè et al., 2018). This discrepancy in findings may be mainly ascribed to the degree of physical development of a particular group of athletes or their exposure to sport-specific tasks. Also, a variety of methods used for balance assessment may play a role in a weak relationship between postural stability and functional movement or athlete performance. While static balance tests may be suitable for shooters, biathletes, or archers, for athletes of freestyle sports, snowboarding, skateboarding, windsurfing, or cycle acrobacy, the dynamic balance tests may represent a more appropriate alternative. Additionally, measurement of CoP variables using laboratory diagnostic systems may not be specific enough for most athletes, namely, those at a high level of competition. Moreover, postural stability may not be a key factor of athletic performance, for instance, a tennis serve or the accuracy and speed in throwing.

### The Role of Neuromuscular Control of Core Stability in Functional Movement and/or Athlete Performance

Analysis of the literature revealed (Table 2) that out of 13 selected studies, seven (54%) investigated the relationship between core (trunk) stability-related variables and functional movement and/or athletic performance (Abt et al., 2007; Nesser et al., 2008; Nesser and Lee, 2009; Chaudhari et al., 2011; Ozmen, 2016; Anand et al., 2017; de Bruin et al., 2021). Three of them (43%) included only variables of athletic performance (Chaudhari et al., 2011; Anand et al., 2017; de Bruin et al., 2021), another three studies (43%) incorporated variables of functional movement and athletic performance (Nesser et al., 2008; Nesser and Lee, 2009; Ozmen, 2016), and one study (14%) focused on changes in the functional movement resulting from compromised core stability (Abt et al., 2007).

**TABLE 2:** Neuromuscular control of core stability and functional movement and/or athlete performance.

Authors	Study design	Participants	Methodology/Main variables	Outcomes	Main findings
**Relationship between core stability and functional movement and/or athletic performance**					
Abt et al., 2007	Changes in pedaling forces and lower extremity joint kinematics as a result of compromised core stability	15 cyclists, members of local road cycling team	3D Motion Analysis System, dependent kinematic variables: total frontal and sagittal plane motion of the hip and knee and total sagittal plane motion of the ankle; Core fatigue: Isokinetic Torso Rotation Test: Biodex System 3 Multi-Joint Testing and Rehabilitation System; Core fatigue workout: 32 min. circuit of 7 exercises targeting core stabilizer muscles	After the core fatigue workout: significant decrease (30.0–43.3%) in peak torque, total work, average power, maximal repetition total work, and average peak torque; an increase in total frontal plane knee motion and total sagittal plane knee and ankle motion (13.4–54.3%); no significant differences for any pedal force or work data	Core fatigue results in altered cycling mechanics that could increase the risk of knee injury; Improved core stability and endurance could promote greater alignment of the lower extremity when riding for extended duration as the core is more resistant to fatigue
Nesser et al., 2008	The relationships between core stability and various strength and power variables in strength and power athletes	29 male football players of the National Collegiate Athletic Association Division I	3 strength variables: 1 RM squat, 1 RM bench press, 1 MR power clean; 4 performance variables: countermovement jump, 20- and 40-yd sprints, 10-yd shuttle run; Core stability: trunk flexion, back extension, and left and right bridge	There is a number of significant but not consistent and weak to moderate correlations between core strength/stability and strength and performance measures	Significant correlations between core strength/stability, even weak to moderate, suggesting that core strength/stability contributes to strength and power performance
Nesser and Lee, 2009	The relationship between core stability and various strength and power variables	16 National Collegiate Athletic Association Division I female football players trained specifically for strength and power	2 strength variables: 1RM squat, 1RM bench press; 3 performance variables: countermovement jump, 10-yd shuttle run, 40-yd sprints; Core stability: trunk flexion, back extension, left and right bridge	There are no significant correlations between core strength/stability and the strength and performance measures	Determination of the effectiveness of core strength or stability requires further research and sport-specific means
Chaudhari et al., 2011	The relationship between lumbopelvic control and pitching performance	48 pitchers who pitched 50 or more innings in Minor League competition of A, AA, or AAA levels	Lumbopelvic control: Level Belt secured to the waist, transition from two-leg to single-leg pitching stance, balance while maintaining a stable pelvic position; Pitching performance: number of innings pitched (IP) during season; Median Level Belt score for the study group 7°	Significantly fewer walks plus hits per inning and significantly more IP during the season in subjects scoring <7° on the Level Belt test than those scoring >7°	Lumbopelvic control influences performance of baseball pitchers; Simple test of lumbopelvic control can identify individuals with better chance of pitching success
Ozmen, 2016	The relationships between core stability, jumping performance and dynamic balance	17 male soccer players	Dynamic balance: Star excursion balance test (SEBT); Core stability: McGill’s protocol; Jumping ability: squat jump test on contact mat	Significant negative correlation between trunk flexion test and jumping height (*r* = −0.705); No significant correlation between side bridge, trunk extension tests and jumping height, and between trunk flexion, side bridge, trunk extension tests and SEBT results	Trunk flexion is associated with squat jump height but not with side bridge and trunk extension tests; Core stability does not contribute to dynamic balance
Anand et al., 2017	The relationship between bowling speed in cricket and core stability	82 cricket medium and medium fast bowlers of district and universities	Core stability: plank test (prone plank, left side plank and right side plank); Bowling speed: BUSHNELL Velocity Speed Gun	There is significant positive correlation between core stability and the bowling speed (*r* = 0.736)	Bowling speed is significantly higher in subjects with well-developed than poorly-developed core stability
de Bruin et al., 2021	The relationship between athletic performance and core stability	83 female athletes from the university teams: hockey (*n* = 24), netball (*n* = 16), running (*n* = 11), soccer (*n* = 15), and tennis (*n* = 17)	Core strength and endurance: Biering-Sørensen tests - isometric back extension (IBE), lateral flexion (LF) and abdominal flexion (AF); Core neuromuscular control (NMC): Welch Allyn FlexiPort pressure biofeedback unit; Athletic performance: T-test, 40 m sprint, medicine ball chest throw (MBCT) and vertical jump (VJ)	Most weak correlations in all sports (*r* = 0.10–0.39); Very strong correlation between VJ and LF (*r* = 0.90); Moderate correlations in all sports between core strength, endurance and motor control and certain athletic performance tests (*r* = 0.40–0.69)	Correlations between core stability and athletic performance are negligible or weak; Athletic performance in different sports is associated with different components of core stability
**Effect of core stability training on functional movement and/or athletic performance**					
Stanton et al., 2004	The effect of short term Swiss ball training (SBT) on core stability and running economy	18 male athletes from Basketball and Touch Football School of Excellence in Sport program: EG (*n* = 8), CG (*n* = 10)	SBT sessions 6 weeks, two times a week, approximately 25 min. during regular training, supervised by researcher; Core stability: 5 level Sahrmann core stability test with Stabilizer Pressure Biofeedback Unit; Maximal aerobic power (VO_2_max) and running economy (RE): incremental treadmill running test to volitional exhaustion	Significant effect of SBT on core stability in the EG; No significant differences in myoelectric activity of abdominal and back muscles, treadmill VO_2_max, RE, or running posture in both EG and CG	SBT has positive effect on core stability without improvements of physical performance
Saeterbakken et al., 2011	The effect of core stabilization training (CST) on maximal throwing velocity	24 female high-school handball players randomly divided into a CST (*n* = 14) and a control group (CG, *n* = 10)	6-week regular handball training in both groups; Additional progressive core stability training program in the CST group (twice a week for 75-min, 6 unstable closed kinetic chain exercises); Throwing velocity: 2 photocell arrays with an accuracy of ± 0.001 s	There is a significant increase of maximal throwing velocity in the CST group (4.9%) but not in the CG	CST using unstable, closed kinetic chain movements improves maximal throwing velocity; Stronger and more stable lumbopelvic hip complex may contribute to higher rotational velocity in multisegmental movements
Sannicandro and Cofano, 2017	The effects of integrative training of core stability on jump performance	44 young basketball players (19 female, 25 male); EG, *n* = 21 (11 female, 10 male), CG, *n* = 23 (11 female, 12 male)	4-week CST in stable and unstable conditions during warm-up (8 sessions, twice a week), followed by basketball drills with CG (60 min); Jump performance: monopodalic jumps (triple hop test, side hop test, and 6m timed hop test) and bipodalic jump (Seargent vertical jump)	Significant improvements in the right and left hop test, the 6m-timed hop left and right test in the EG; A significant improvement in vertical jump in the CG	Core stability program is effective in improving monopodalic jump ability in prepubertal basketball players
Vitale et al., 2018	The effects of neuromuscular training program on dynamic balance and vertical jump performance	24 elite junior male skiers randomized in an experimental group (EG, *n* = 12) and a control group (CG, *n* = 12)	8-week training program (16 sessions, 3 phases); partly different exercises on core stability, body-weight strengthening and plyometric exercises on dynamic postural control and vertical jump performance in each phase; circuit training form during warming up (30 min); Dynamic balance: lower quarter Y-Balance test (YBT) with standardized testing protocol; Jumping performance: countermovement (CMJ) and drop jump (DJ) on Optojump Next	Positive effects on pre to post measures in anterior, postero-medial, postero-lateral directions, and composite YBT score for both lower limbs in the EG; No significant changes in the CG; No significant changes in CMJ and DJ in both EG and CG	There is a positive effect of neuromuscular training on dynamic balance ability but not on vertical jump performance; It may be effective in increasing lower limb joint awareness and postural control
Kuhn et al., 2019	The effects of core stabilization training (CST) on maximal throwing velocity and core strength parameters	20 female handball players from German non-elite handball squad	6-week CST (twice a week for 45 min., 9 specific core and rotational exercises for ventral, dorsal and lateral core muscles chain on an unstable surface); Maximum voluntary isometric strength (MIS) of the trunk using isometric dynamometer Beck-check 607; Endurance strength of ventral, dorsal and lateral core chains using a Swiss Olympic Medical Center core performance test battery; Throwing velocity using OPTOJump Next	A significant improvement in MIS of left lateral core muscle chain in the EGcompared to the CG; A significant improvement in MIS of ventral core endurance (35%) and the lateral right core muscles (21%) in the EG; A significant increase in throwing velocity of jump throw in both EG (12%) and CG (8%) but not velocity of standing throw	CST effectively increases isometric strength and endurance of core muscles but does not enhance throwing velocity when compared to standard training
Felion and DeBeliso, 2020	The effect of core training (CT) program on force production in torsional movements	Students, members of baseball team at Granger HS, UT, United States	Experimental group (EG): 6-week CT program (twice a week, 1 h/day), in addition to specific training; Control group (CG): 6-week baseball specific training only (twice a week, 2 h/day). Throwing velocity (TV) and ball-exit velocity (BEV) using Stalker Sport II radar gun; BEV: speed of the ball immediately after being struck by the baseball bat	Neither EG nor CG increase in TV following the 6-week CT intervention; A significant increase in BEV in the EG but not in the CG	Implementing of CT with additional rotational exercises with free weights, resistance bands, or medicine balls leads to additional gains in torso rotational strength and potentially improvement in BEV

The remaining six studies (46%) evaluated the effects of various core or neuromuscular training programs on core stability, functional movement, and athletic performance (Stanton et al., 2004; Saeterbakken et al., 2011; Sannicandro and Cofano, 2017; Vitale et al., 2018; Kuhn et al., 2019; Felion and DeBeliso, 2020). Three of them (50%) investigated the effects of core stabilization exercises on functional movement and performance variables, strength, or core stability (Stanton et al., 2004; Sannicandro and Cofano, 2017; Kuhn et al., 2019). Two studies (33%) examined the effects of core stabilization exercises only on variables of athletic performance (Saeterbakken et al., 2011; Felion and DeBeliso, 2020). One study (17%) evaluated the effect of neuromuscular training on selected parameters of functional movement (Vitale et al., 2018).

Regarding the sport, eleven studies (85%) were conducted in team sports, such as baseball, basketball, cricket, football, handball, soccer, and touch ball (Stanton et al., 2004; Nesser et al., 2008; Nesser and Lee, 2009; Chaudhari et al., 2011; Saeterbakken et al., 2011; Ozmen, 2016; Anand et al., 2017; Sannicandro and Cofano, 2017; Kuhn et al., 2019; Felion and DeBeliso, 2020; de Bruin et al., 2021) and two studies (15%) were carried out in individual sports, such as cycling and skiing (Abt et al., 2007; Vitale et al., 2018).

#### The Relationship Between Core Stability and Functional Movement and/or Athletic Performance

Among seven studies, six investigated the association of core stability with variables of athletic performance (Chaudhari et al., 2011; Anand et al., 2017; de Bruin et al., 2021) or both functional movement and athletic performance (Nesser et al., 2008; Nesser and Lee, 2009; Ozmen, 2016), whereas one study dealt with changes in functional movement resulting from compromised core stability (Abt et al., 2007).

The most investigated characteristics of core stability (Nesser et al., 2008; Nesser and Lee, 2009; Ozmen, 2016; Anand et al., 2017) were core or lumbopelvic neuromuscular control (Chaudhari et al., 2011; de Bruin et al., 2021), and core strength and endurance (Abt et al., 2007; de Bruin et al., 2021). Among functional movement characteristics, it was the kinematics of movement (Abt et al., 2007) and jumping abilities that stood out (Ozmen, 2016; de Bruin et al., 2021), whereas factors of athletic performance included ball speed (Anand et al., 2017), running speed, agility, and explosiveness of upper body (de Bruin et al., 2021). All studies dealing with the association of core stability with functional movement and athletic performance used a cross-sectional design. In all seven studies, only one selected group of athletes of a certain type of sport was tested.

Regarding the methodology of core stability characteristics, the following tests were used: trunk flexion, back extension, left and right bridge (Nesser et al., 2008; Nesser and Lee, 2009), core stability McGills protocol (Ozmen, 2016), prone plank, left and right side plank (Anand et al., 2017), and Biering-Sørensen test (de Bruin et al., 2021). The “Level belt” was used for the lumbopelvic control (Chaudhari et al., 2011), isokinetic torso rotation test on a Biodex system and 32 min circuit of exercises targeting the core muscles evaluated core muscle fatigue (Abt et al., 2007), and biofeedback unit was applied for the core neuromuscular control (Ozmen, 2016). Regarding the functional movement characteristics, three-dimensional motion analysis (Abt et al., 2007) and star excursion balance test for dynamic balance (Ozmen, 2016) were used. Athletic performance characteristics were evaluated using the radar speed gun (Anand et al., 2017), 20-m run, 40-m run, *T*-test, agility test, shuttle run, medicine ball throw (Nesser et al., 2008; Nesser and Lee, 2009; de Bruin et al., 2021), squat jump (Ozmen, 2016), and the number of innings pitched during a season (Chaudhari et al., 2011).

Core stability provides a foundation for force production in the lower and upper limbs (Willardson, 2007). This is a requisite for optimal functional movement and consequently also for better athletic performance (Abt et al., 2007; Chaudhari et al., 2011; Anand et al., 2017). However, some studies do not find this link between core functions and the movement of lower and upper limbs. In general, two research approaches exist that examine the association of core stability (lumbopelvic control) with functional movement and athletic performance. Some studies examined the importance of core stability or lumbopelvic control using strength, endurance, agility, speed, or other physical abilities tests as surrogate measures of functional movement and athletic performance (Nesser et al., 2008; Nesser and Lee, 2009; Ozmen, 2016; de Bruin et al., 2021). It has been proposed that well-trained athletes have general level of abilities, such as agility, explosive power, and speed, in addition to core stability, regardless of the specificity of the sport, and that there is a relationship between them. Other studies used direct measures of functional movement or athletic performance (Abt et al., 2007; Chaudhari et al., 2011; Anand et al., 2017). For instance, investigating the relationship between cycling mechanics and core stability revealed that improved core stability and endurance could promote greater alignment of the lower extremity when riding for extended durations as the core is more resistant to fatigue (Abt et al., 2007). Lumbopelvic control influences overall performance for baseball pitchers, thus a simple test of lumbopelvic control can potentially identify individuals who have a better chance of pitching success (Chaudhari et al., 2011). Throwing accuracy is significantly better in cricket bowlers with well-developed than poorly developed core stability (Anand et al., 2017).

The association of core stability with some variables of athletic performance in sports such as hockey, netball, tennis, soccer, and running supports the fact that its significance regarding some motor abilities in particular sports partly differs. However, when these sports were analyzed separately, there were similar moderate correlations between core strength or endurance and motor abilities in the tests used (de Bruin et al., 2021). There were strong correlations between abdominal flexion endurance and the vertical jump in runners, and between isometric back extension strength and the sprint in tennis players. However, the core strength and/or stability does not correlate with the strength and performance measures (10 yard shuttle run, 40 yard sprint, countermovement jump, 1RM squat, 1RM bench press) in athletes who train for strength and football skills. Despite these non-significant correlations, it is not reasonable to neglect the core. Nonetheless, it seems that the core musculature is no more important than any other part of the body (Nesser and Lee, 2009).

A belief that core stability is important for strength and power production in sport was not corroborated in male football players. The core stability was significantly but not strongly correlated with power and strength variables (10-yd shuttle run, 20- and 40-yd sprints, countermovement jump, 1RM squat, 1RM bench press, 1RM power clean). Correlations between core stability and strength or power, and sprints or shuttle run were moderate to weak but significant. This indicates that core strength contributes to power and strength performance and therefore should be taken into account (Nesser et al., 2008). However, there was a negative correlation between the jump height and trunk flexion, and no significant association was found between the jump height and side-bridge trunk extension in male soccer players. Similarly, the relationship between core stability and dynamic balance was not significant (Ozmen, 2016). These findings indicate that an understanding of the role of core stability in body movements most likely requires testing under sport-specific conditions. All of the athletic performance measures were mainly one repetition of explosive movements or sprints lasting a few seconds. The core stability was evaluated using isometric muscle contractions or muscle endurance tests. However, the core stability and performance of these two variables should not be compared. While sub-maximal muscle contractions, activation of more slow-twitch muscle fibers, and anaerobic glycolysis are typical for most core stability tests, the agility, power, strength, and running tests involve primarily maximum force production, activation of fast twitch muscle fibers, and the ATP–CP energy system (Nesser et al., 2008; Nesser and Lee, 2009; de Bruin et al., 2021).

The second type of study represents relationships between core stability and bowling speed in cricket, walks, hits per innings pitched and total innings pitched in baseball and functional movement in cycling. Cricket players with well-developed stability of the core manifest high quality in the kinetic chain of movements when bowling that probably results in increased bowling speed. They can better control their trunk position and motion over the pelvis and leg. This allows optimum generation and transfer of force to the terminal segment in the kinetic chain of specific movements. Core stability provides integration of proximal and distal segments in increasing bowling speed (Anand et al., 2017). Lumbopelvic control is related to performance in baseball pitchers (Chaudhari et al., 2011). The study revealed differences between lumbopelvic control and walks plus hits per innings pitched and total innings pitched. Significantly lower walks plus hits per innings pitched were found in the group with better than those with poorer lumbopelvic control. Furthermore, core stability also plays a role in the functional movement in cycling. For instance, lower extremity cycling mechanics is influenced by the core fatigue workout. Several kinematic variables were altered whereas work variables and the pedal force remained unchanged (Abt et al., 2007).

#### Core Stability Training and Functional Movement and/or Athletic Performance

Training programs were usually aimed at the increase of athletic performance factors (Saeterbakken et al., 2011; Kuhn et al., 2019) or variables of functional movement (Stanton et al., 2004; Sannicandro and Cofano, 2017; Vitale et al., 2018; Felion and DeBeliso, 2020) and were often combined with the development of core stability and strength (Stanton et al., 2004; Kuhn et al., 2019; Felion and DeBeliso, 2020) or the dynamic balance (Vitale et al., 2018).

The duration of intervention was from 4 to 6 weeks (Stanton et al., 2004; Saeterbakken et al., 2011; Sannicandro and Cofano, 2017; Kuhn et al., 2019; Felion and DeBeliso, 2020) or 8 weeks (Vitale et al., 2018), two times per week with a duration of 25–45 min (Stanton et al., 2004; Sannicandro and Cofano, 2017; Vitale et al., 2018; Kuhn et al., 2019) to 60–75 min (Saeterbakken et al., 2011; Felion and DeBeliso, 2020). While the 25–30 min programs were a part of warming up, the 45–75 min programs were organized apart from standard training. Core stabilization training programs were supervised by coaches, conditioning specialists, or researchers.

Core stabilization training programs included core exercises (Stanton et al., 2004; Saeterbakken et al., 2011; Sannicandro and Cofano, 2017; Kuhn et al., 2019; Felion and DeBeliso, 2020), or core exercises combined with plyometrics and body strengthening (Vitale et al., 2018). These exercises were often performed in unstable conditions or in both stable and unstable conditions (Stanton et al., 2004; Sannicandro and Cofano, 2017; Vitale et al., 2018; Kuhn et al., 2019).

The assessment of athletic performance or measurement of functional movement variables was focused on throwing velocity (Saeterbakken et al., 2011; Kuhn et al., 2019; Felion and DeBeliso, 2020) and jumping performance (Vitale et al., 2018). Running economy was assessed with a test to exhaustion on a treadmill (Stanton et al., 2004). The core assessment included the Swiss Olympic test (Kuhn et al., 2019) and the Sahrmann core stability test (Stanton et al., 2004); dynamic balance was tested by means of Y-Balance test (Vitale et al., 2018); and jump abilities by using the side hop test, triple hop test, 6-m timed hop test, and the Sargent vertical jump test (Sannicandro and Cofano, 2017).

Core stability and core strength training have been used for the improvement of functional movement and consequently also athletic performance. The purpose of core stability exercises is to control the lumbar spine, whereas core strength exercises improve the transfer of muscle power, activation of local stabilizers, and global mobilizers (Saeterbakken et al., 2011; Sharrock et al., 2011; Sannicandro and Cofano, 2017). The training programs incorporating core stability exercises performed under stable or unstable conditions showed improvements in core muscle strength, muscular endurance, and body balance (Stanton et al., 2004; Vitale et al., 2018; Kuhn et al., 2019). However, there is a controversy as to whether an increase in core stability and strength is transferred to athletic performance.

For instance, a 6-week isolated resistance training program in young baseball players did not improve the throwing velocity in contrast to the ball-exit velocity (Felion and DeBeliso, 2020). Similar findings were found also after a 6-week core stabilization training in adult female handball players. Both experimental and control groups significantly increased throwing velocity of the jump throw, but their throwing velocity of the standing throw remained unchanged (Kuhn et al., 2019). Furthermore, an integrated short-term Swiss ball training failed to enhance the running economy measured by VO_2_max, vVO_2_max, or running economy at speeds of 60, 70, 80, or 90% vVO_2_max (Stanton et al., 2004). On the other hand, an isolated progressive core stability training in unstable conditions improved the throwing velocity significantly in young handball players (Saeterbakken et al., 2011). Also, an 8-week integrated neuromuscular training focused on core stability, plyometrics, and dynamic postural control led to an improvement of postural stability but not of jump performance in junior alpine skiers (Vitale et al., 2018). Furthermore, a 4-week integrated core stabilization program improved the one-leg jump abilities but not the bipedal vertical jump in the prepubertal athletes (Sannicandro and Cofano, 2017).

Depending on how the special core stabilization programs were integrated into standard training, it is possible to distinguish two variants, that is, either integrated programs conducted during warm-ups within a training session (Stanton et al., 2004; Sannicandro and Cofano, 2017; Vitale et al., 2018; Kuhn et al., 2019) or isolated additional programs carried out as the standard training (Felion and DeBeliso, 2020; Saeterbakken et al., 2011). With regard to these differences in integrated and isolated core stabilization training programs, findings in the literature are not consistent.

Most studies investigated the association of core stability with functional movements and athletic performance or the effect of specific core stabilization programs on functional movement and athletic performance in junior or younger age groups (Stanton et al., 2004; Nesser et al., 2008; Nesser and Lee, 2009; Chaudhari et al., 2011; Saeterbakken et al., 2011; Anand et al., 2017; Sannicandro and Cofano, 2017; Vitale et al., 2018; Felion and DeBeliso, 2020; de Bruin et al., 2021). However, only a few studies have investigated the role of core stability in the functional movement and athletic performance in adult athletes and the changes induced by core stabilization training (Abt et al., 2007; Chaudhari et al., 2011; Ozmen, 2016; Kuhn et al., 2019). The reason for this disproportionality could be the accessibility of young athletes compared to the elite ones for participating in intervention studies.

### Limitations in the Current Studies Investigating the Relationship Between Postural and/or Core Stability and Athlete Performance and Proposals for Further Research

An analysis of the literature revealed several gaps in the existing studies (Table 3). There is still a lack of research that seeks to investigate the relationship of body balance and stability of the core with sport-specific performance. Although the importance of the core musculature for spine stabilization and postural control has been emphasized during the past decade, the supporting evidence is still scarce. Recently, increased research efforts have been accomplished to investigate effective exercise programs for improving spinal stability and body balance. Practitioners suggest that a strong core could contribute to better balance and proper posture with a positive impact on increasing their athletic performance and/or decreasing the occurrence of back pain. While postural and core stability may be a key factor in the prevention of musculoskeletal disorders, it seems that much less evidence exists on their role in sport-specific performance and related functional movements. These gaps revealed in the literature should be addressed in future studies.

**TABLE 3:** Research gaps identified in the literature and suggestions for future studies.

Gaps and limitations revealed in the literature	Suggestions for future studies
There is a lack of studies investigating the relationship between core stability or core strength and functional movement and/or athletic performance. There is still inconsistent definition of core stability and core strength in spite of an increased number of studies in this field of research. The research in this field has been conducted mainly in shooting, biathlon, archery, gymnastics, and team sports. More research has been carried out in team than individual sports. Small sample sizes occur in most studies, which could reduce its power and increase the margin of error. There are only few studies conducted on competitive athletes of a high performance level. The analysis of the literature in this scoping review related to the role of core stability in functional movement and/or athlete performance revealed that nine studies (69%) were conducted with regularly competing high school or university athletes or athletes from lower competitions (Stanton et al., 2004; Abt et al., 2007; Chaudhari et al., 2011; Saeterbakken et al., 2011; Ozmen, 2016; Anand et al., 2017; Kuhn et al., 2019; Felion and DeBeliso, 2020; de Bruin et al., 2021), three studies (23%) with young elite athletes (Nesser et al., 2008; Nesser and Lee, 2009; Vitale et al., 2018), and one study (8%) with very young athletes (Sannicandro and Cofano, 2017). Regarding the age of participants, eight studies (62%) included adult athletes (Abt et al., 2007; Nesser et al., 2008; Nesser and Lee, 2009; Chaudhari et al., 2011; Ozmen, 2016; Anand et al., 2017; Kuhn et al., 2019; de Bruin et al., 2021) and five studies involved young athletes (Stanton et al., 2004; Saeterbakken et al., 2011; Sannicandro and Cofano, 2017; Vitale et al., 2018; Felion and DeBeliso, 2020). There is a lesser number of research studies conducted on female than male participants. Regarding gender, the analysis of the literature related to the role of core stability in functional movement and/or athlete performance revealed that eight studies (62%) included male athletes (Stanton et al., 2004; Abt et al., 2007; Nesser et al., 2008; Chaudhari et al., 2011; Ozmen, 2016; Anand et al., 2017; Vitale et al., 2018; Felion and DeBeliso, 2020), four studies (31%) female athletes (Nesser and Lee, 2009; Saeterbakken et al., 2011; Kuhn et al., 2019; de Bruin et al., 2021), and one study (7%) both girls and boys (Sannicandro and Cofano, 2017). The control group is rarely included. Non-sporting population cannot be included in most of the studies because of the athletic performance tests used. There is a missing information on the level of athlete performance. General physical fitness tests rather than sport-specific tests are used. Variables analyzed are not precisely described. Experiments are conducted in different training periods (pre-season, in-season, post-season) what limits a comparison of findings. The average duration of training programs is from 4 to 8 weeks while the training sessions vary from 25 to 30 min, in some cases even from 60 to 75 min, which very often combine balance, strength and core muscle exercises. There is insufficient analysis of neuromuscular mechanisms underlying significant associations of postural and core stability with functional movement and/or athlete performance. Balance, strength, plyometric or endurance exercises are usually associated with training induced improvements of postural and core stability but not with athlete performance.	In comparison with balance research, more attention should be paid to investigations related to the role of core stability and core strength in functional movement and/or athletic performance. The authors should use uniform terminology of core stability and core strength based on their characteristics, similarly as it is in the case of balance research. As core stability and strength represent an integral part of athlete performance in sports based on lifting tasks and trunk rotations, their role in performance in acrobatic, combat, power and water sports should also be investigated. The number of participants in research studies should be increased. The research studies should include more elite athletes. In such a case, adults should be preferred over young participants. The number of female participants should be increased. The control group should be included, especially in intervention studies. Information on the degree of physical development of athletes and their exposure to sport-specific tasks should be included. Better understanding the role of postural and core stability in athletic performance requires testing under conditions specific to a particular sport. Therefore, testing under sport-specific conditions should be preferred, particularly in athletes at a high level of competition. Corresponding variables should be better specified in relation to functional movements in sports with high demands on postural and core stability. Studies investigating the association of postural and core stability with functional movement and performance in athletes should be carried out during a period of their high level of sport-specific skills. The duration of training sessions and training programs should be separately specified for balance exercises and core stabilization or core strengthening exercises. Greater attention should be paid to the interpretation of findings. Further research is needed to investigate the relationship between postural or core stability and sport-specific performance and their changes after sport-specific training.

## Conclusion

This scoping review revealed that among a variety of studies investigating the role of neuromuscular control of postural and core stability in functional movement and/or athlete performance, only a few revealed the relationships between them. Postural stability was found to play an essential role in performance in archery, biathlon, gymnastics, shooting, and team sports (e.g., basketball, hockey, soccer, tennis). Also, core stability and strength represent an integral part of athlete performance in sports based on lifting tasks and trunk rotations. Variables of these abilities are associated with performance-related skills in cricket, cycling, running, and team sports (e.g., baseball, football, hockey, netball, soccer, tennis). Better neuromuscular control of postural and core stability contribute to more efficient functional movements specific to particular sports. Training programs incorporating general and sport-specific exercises that involve the use of postural and core muscles showed an improvement of body balance, back muscle strength, and endurance. However, there is controversy about whether the improvement in these abilities is translated into athletic performance. Identifying the relationship of passive, active, and neural mechanisms underlying balance control and spinal posture with athlete performance would provide a basis for a multifaced approach in designing training and testing tools addressing postural and core stability in athletes under sport-specific conditions.

## Author Contributions

Both authors have made a substantial, direct, and intellectual contribution to the work, and approved it for publication.

## Conflict of Interest

The authors declare that the research was conducted in the absence of any commercial or financial relationships that could be construed as a potential conflict of interest.

## Publisher’s Note

All claims expressed in this article are solely those of the authors and do not necessarily represent those of their affiliated organizations, or those of the publisher, the editors and the reviewers. Any product that may be evaluated in this article, or claim that may be made by its manufacturer, is not guaranteed or endorsed by the publisher.
